# A Review of Gas Measurement Practices and Sensors for Tunnels

**DOI:** 10.3390/s23031090

**Published:** 2023-01-17

**Authors:** Jorge J. Cepa, Rubén M. Pavón, Paloma Caramés, Marcos G. Alberti

**Affiliations:** Departamento de Ingeniería Civil: Construcción, E.T.S de Ingenieros de Caminos, Canales y Puertos, Universidad Politécnica de Madrid, 28040 Madrid, Spain

**Keywords:** sensor, gas measurement, tunnel

## Abstract

The concentration of pollutant gases emitted by traffic in a tunnel affects the indoor air quality and contributes to structural deterioration. Demand control ventilation systems incur high operating costs, so reliable measurement of the gas concentration is essential. Numerous commercial sensor types are available with proven experience, such as optical and first-generation electrochemical sensors, or novel materials in detection methods. However, all of them are subjected to measurement deviations due to environmental conditions. This paper presents the main types of sensors and their application in tunnels. Solutions will also be discussed in order to obtain reliable measurements and improve the efficiency of the extraction systems.

## 1. Introduction

The growing concern about indoor air quality [1,2,3], the emission of gases in industrial processes and transport [4,5,6], as well as the stringency of new regulations have led to further research focused on the development of effective sensors in gas measurement. Tunnel-type infrastructures have always been affected by gas concentration levels, so monitoring these levels has important applications in their operation and maintenance (O&M). Road tunnels support high levels of combustion gases, such as carbon oxides (COx), nitrogen (NOx), and particulate matter (PM), or organic compound species, such as volatile organic compound (VOC), or hydrocarbons (HCs), such as BTEX (benzene, toluene, ethylbenzene and xylene) [7,8,9,10,11,12,13,14,15,16,17,18]. Keeping adequate concentration levels of these gases is essential for their facility management (FM). High levels of pollutants affect health, so they should be kept at low values, it being advisable to keep them in the region of 400 ppm of CO_2_ in the domestic sphere, although these levels may be higher in the case of industrial facilities, as regulated by Spanish legislation [19]. The presence of these pollutants also affects the structure, accelerating degradation mechanisms, such as the carbonation of the concrete [16].

Monitoring the air quality inside the tunnel in real time is essential in order to be able to activate the ventilation system. The volume of air extracted by this fan system must be one which maintains tolerable levels of gases without incurring high operating costs, which are mainly due to the amount of energy used during its functioning. However, studies quantifying these costs are scarce. Some researchers have even reported that up to 75% of operating costs come from ventilation [20,21,22]. In addition, the continued use of exhaust fans leads to the accelerated wear and tear of their moving parts, resulting in a shortened service life and, consequently, further costs associated with their replacement [23]. In order to mitigate these high costs, some researchers propose the use of natural ventilation in long tunnels, which can lead to savings of up to 40% in smoke extraction costs [20,21,24].

Air quality monitoring can be performed from different approaches, depending on the chosen gas and sample measurement method. Within the gas mixture inside the tunnel, PM, CO, CO_2_, NO_2_, or BTEX have traditionally been measured [15,16,17], although other components of the gas mixture, such as oxygen (O_2_) [25] or VOC, can also be monitored [18,26]. Numerous types of sensors for gas detection are available on the market. They can be mainly classified as either optical sensors or electrochemical sensors based on measuring method [27,28,29]. Each of them has advantages and limitations, such as working temperature, its useful life, or response time. External factors, such as temperature, humidity, or the presence of other gases in urban atmospheric environments produce deviations in the measurement that are aggravated over time, regardless of the type of sensor [30,31,32]. Therefore, the regular recalibration of measurement systems is necessary to obtain reliable measurements.

Concern about indoor air quality has increased in recent years, leading to the proliferation of low-cost sensors for measuring indoor CO_2_ concentration levels [33]. Research and advances in this type of sensor, linked together and periodically recalibrated, can be extrapolated to the control of gas levels in tunnels, leading to a significant leap in the reduction in ventilation operating costs.

The following sections compare the main sensors used based on the published literature, focusing on their application for measuring gases in tunnels, as well as the issues in this type of infrastructure. Additionally, some new research with new materials and their possible applications in the sensorization of this type of structure will be presented. 

## 2. Types of Sensors

Various approaches are currently known for gas detection, such as sensor type optical, semiconductor, catalytic, thermometric, photoacoustic, chemiluminescent, gas chromatographic and others [27,34]. Among detection methods, electrochemical sensors and optical non-dispersive infrared (NDIR) detectors are widely used in measurement technology [35]. This article focuses on the analysis of these two detection methods in sensors. 

Furthermore, among the measurement parameters, there are several concepts to consider, such as sensitivity, selectivity, working temperature range, stability of the measurement under working conditions or lifetime. Sensitivity is related to the detection limit, which is a measure of the smallest concentration that can be determined with a specified accuracy or reproducibility. Selectivity refers to the ability of the sensor to determine the concentration of a specific gas in the presence of other gases. These concepts will be key to determining the capabilities of each detection method and their potential application in real-time monitoring of gas concentration inside a road tunnel.

### 2.1. NDIR Sensors

**Figure 1:**
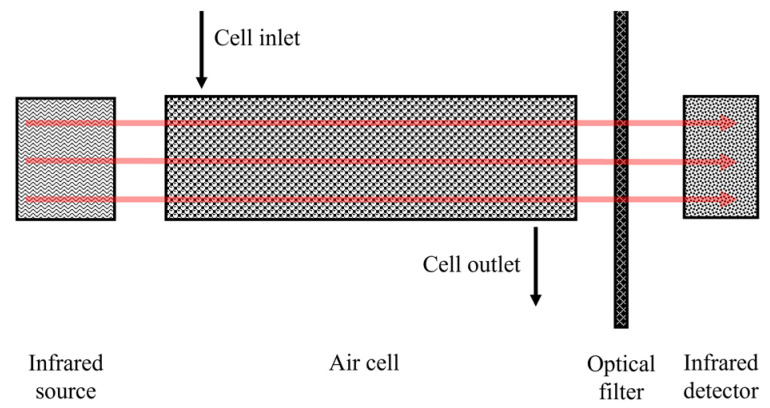
Diagram of an NDIR-type sensor operation based on [36].

NDIR gas measurement falls into the category of optical sensors. This is an application of infrared spectroscopy. The NDIR technique for gas measurement aims at absorbing wavelengths in the infrared spectrum as a way to identify particular gases [36]. A simple NDIR gas sensor consists of an infrared emitter, a detector, an optical filter, a gas cell and circuit elements for signal processing [37,38]. The operation of this type of sensor is shown in Figure 1.

This type of sensor is the most widely used nowadays, with a large variety of simple devices on the market. The main advantage of NDIR sensors is their longer lifetime, which is more than 10 years, compared to other detection methods. However, they suffer from several problems, such as selectivity due to interference from nearby gases, sensitivity due to small signal output from weak gas absorption and the elevated cost owing to highly sophisticated optical components [39,40,41,42,43]. This sensitivity is determined by the detection limit, which depends on both the signal strength (or sensitivity) and the signal stability (signal-to-noise ratio). As such, they tend to have high power consumption, which makes them difficult to use in battery-powered devices for in situ monitoring applications. In addition, spectroscopic methods are voluminous and require a long response time for high concentrations [44]. 

**Figure 2:**
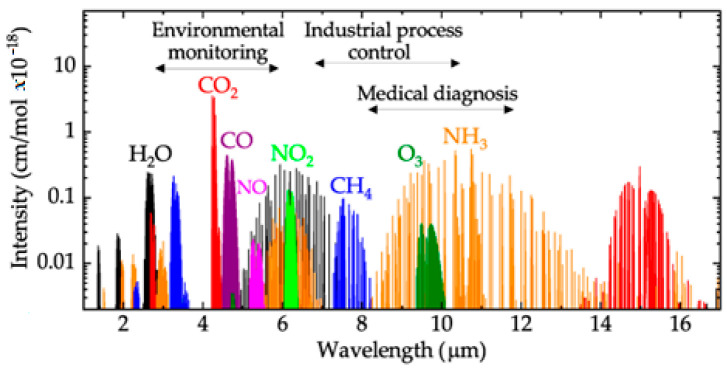
Midinfrared absorption spectra of selected molecules with their relative intensities. H_2_O water; CO_2_: carbon dioxide; CO: carbon monoxide; NO: nitric oxide; NO_2_: nitrogen dioxide; CH_4_: methane; O_3_: oxygen; NH_3_: ammonia [46].

The interference problem is between gas adsorption bands, such as the spectral signature of NO_2_ with water vapor shown in Figure 2. This can be mitigated with new optics techniques, although it has not yet been fully resolved. In addition, the presence of water vapor could affect other sensor electronics or cause artefacts to form for the target gases, distorting the measurement [36,43,45]. The water vapor problem can be avoided by operating the gas cell of the NDIR sensor at high temperatures, but this usually results in the generation of other artefacts, causing a measurement error.

More recent studies have shown the advantages of this type of sensor under ambient conditions. In addition, there has been an increasing need to measure indoor air quality, proliferating and making this type of sensor cheaper and extending its use to the domestic environment, where concentrations should not exceed 1000 ppm and the ambient temperature is around 20 °C and relative humidity is 50% [33,42,47]. They have also been used to measure atmospheric air quality by studying the influence of pressure, temperature and humidity on CO_2_ measurement [48,49].

### 2.2. Electrochemical Sensors

Within electrochemical sensors there are a multitude of device methods with different materials. They usually consist of at least two electrodes; one sensing or working and one reference electrode. These electrodes are connected through a thin layer of electrolyte [27]. Viable alternatives, such as sensors based on solid oxide electrolytes have been appearing recently. Such sensors are considered the most reliable way to detect CO_x_ in industrial applications. These sensors operate at temperatures of 450 °C and above to ensure sufficient ionic conductivity in a solid oxide electrolyte [29,50]. 

There are also electrochemical gas sensors in solution. These are characterized by high complexity, high sensitivity and stability of the sensor and work at room temperature [25,51,52,53,54,55]. Sensors can be found in aqueous solution, although other types of solutions are usually experimented with to improve the properties of the sensor and to reduce the dependence of the sensor on the pH of the solution. These types of sensors are often used in very specific engineering or medical applications, such as wastewater treatment [55] or clinical blood gas analysis, respectively [52].

**Figure 3:**
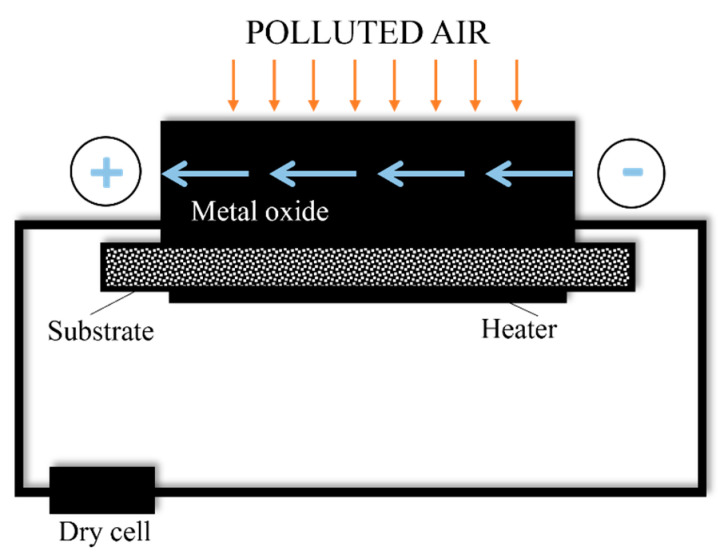
Diagram of MOS-type sensor operation. Based on [34].

Additionally, there are sensors based on metal oxide semiconductors (MOS). The detection principle of semiconductor-type sensors is based on the change of the electrical resistance of the MOS, depending on the composition of the gaseous atmosphere [56,57]. The schematic of this type of sensor is shown in Figure 3.

This type of sensor has good sensitivity, a fast response time and stable performance and is the most cost-effective [58,59,60,61]. However, the sensor signal can be affected by the adsorption of different components of the gaseous atmosphere on the semiconductor surface, such as other gases with atmospheres or humidity, which affects the selectivity [28,50,62]. In addition, such sensors typically have optimum operating temperatures of several hundred degrees Celsius [29,41,63,64]. 

Another material for chemical gas detection is polymer-based sensors [44]. This type of sensor has a short response time and an operating range at ambient temperature [65]. However, they have poor selectivity, as well as short- and long-term sensor deviation, which lead to inaccurate measurements over time [66,67]. 

Recently, new sensing materials, such as carbon nanotubes or graphene, have appeared [25,68,69,70,71,72]. Even though a lot of research has been carried out on these sensors, there are still some limitations which need to be addressed and rectified to improve the efficiency in the utilization of these sensors on a commercial basis [72]. 

**Figure 4:**
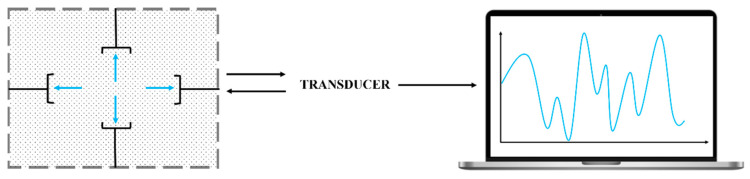
Schematic diagram of operation of MOF-type sensors. Based on: [66].

Metal–organic frameworks (MOFs) have arisen as a promising option in the gas sensor industry. Such sensors are fabricated by assembling metal nodes with organic linkers [73,74,75,76,77,78]. MOFs sensors have different working principles, such us refractive index sensing [78,79,80], chemical resistance [81,82], mass sensitive mode [77,83], electrochemical impedance spectroscopy [67,84], responsive fluorescence [74,75,85] or variations in the luminescence properties [86]. Their high surface-area-to-volume ratio is especially beneficial for sensing applications, as it increases the possibility of interaction between sensing materials, leading to high sensitivity, as well as having high thermal and chemical stability [67,74,75,85]. Sensors require two main components: a sensing layer and a transducer, as shown in Figure 4. The sensing layer interacts selectively with the target analytes, so that various changes in the physicochemical properties of the system (e.g., capacitance, mass, conductivity, optical properties, etc.) are detected, and the transducer translates the changes into measurable signals.

MOF-type sensors have a promising future for selective gas detection due to their sensitivity, wide operating temperature range and structural and chemical adaptability [66,87]. However, although there is increasing research and diversity in these types of compounds and transducers, most investigation is focused on developing laboratory prototypes for CO_2_ detection, leaving aside the rest of the gases. Another problem that has arisen is the tendency of some of these materials to hydrolyse in humid conditions. Some authors have solved this problem by using materials that are stable in water, at the cost of losing sensitivity in favour of longer durability at room temperature [88].

### 2.3. Comparison between Sensors

All current technologies for gas detection, and especially CO_2_, have their own set of advantages and limitations that make them relevant for specific detection applications. Table 1 gives a published comparison of the main characteristics of the most used sensor types. 

This comparison excludes the most recent developments. For example, in the case of optical sensors, numerous NDIR sensors have been developed and commercialized at lower costs, making them widely used in many domestic and industrial applications [9,48,90]. Response times for electrochemical and infrared absorption sensors are also described as poor, but recent studies report response times of less than 1 min for concentrations below 2000 ppm [27,42,43,45]. Although the selectivity of this type of sensor is rated as “excellent” in the comparative table, several authors identify difficulties in the selectivity of this type of sensor [39]. Furthermore, this comparison fails to cover some relevant aspects, such as the working temperature (very high in electrochemical sensors), or the influence of environmental factors, such as temperature and humidity. 

Furthermore, there are nowadays a multitude of devices on the market for each measuring method. This results in a wide variety of sensors whose parameters, in terms of sensitivity, selectivity, response time or cost, cover the specific needs for each case. In addition, advances in data processing can predict trends in air quality. This can lead to more reliable measurements with lower capacities of sensors.

### 2.4. Sensor Calibration

**Figure 5:**
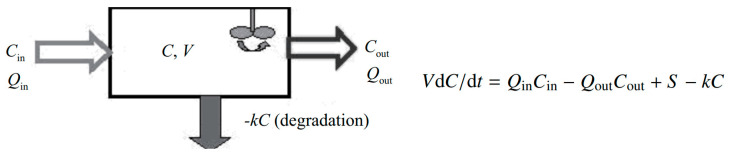
Schematic diagram of a fully mixed box model. *C*_in_, *C* and *C*_out_ are concentrations of the monitored gas in the inflow, indoor air and outflow, respectively, *Q*_in_, *Q*_out_ are air flows into/out of the building/space *V* is room volume [32].

Gas monitoring is becoming increasingly relevant in urban environments. It is used to measure air quality, both for air pollution in cities and indoor gas concentrations. CO_2_ sensors are often used to keep ventilation controlled on demand. However, the environment in which the measurement is taken is susceptible to changes in ambient conditions that result in erroneous measurements, regardless of the type of sensor. Gas concentration in a space depends on the concentration of the in-flowing air, the concentration of the out-flowing air and the internal generation rate of the gas in the space minus the degradation of the gas, as shown in Figure 5 [32].

**Figure 6:**
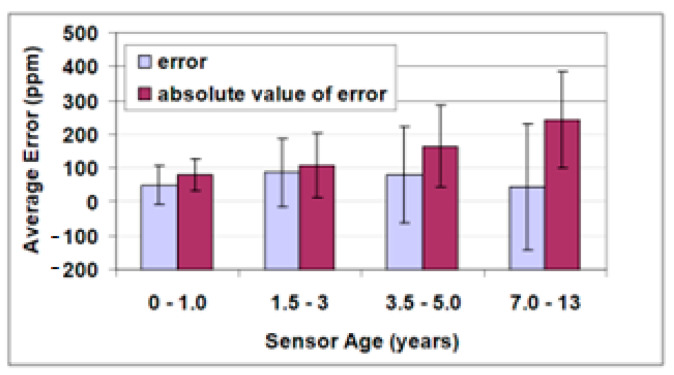
Error from single-concentration calibration checks and multi-concentration calibration challenges at 510 ppm plotted versus sensor age. The error bars represent one standard deviation in the error [91].

Proper demand-controlled ventilation requires accurate CO_2_ measurements. However, some research has reported substantial measurement errors. In the case of infrared sensors, no association between sensor age and measurement accuracy can be established, as shown in Figure 6, and neither can these inaccuracies be attributed to errors in the translation of the sensor output signal. This research at malls in the USA determined that, in most cases, no calibration was performed during the lifetime of the sensor after the initial factory calibration.

External factors, such as temperature, humidity or the presence of other gases in urban atmospheric environments produce deviations that are aggravated over time and affect each type of sensor differently [30,31]. For example, for MOS-type sensors, the electrical conductivity of the sensor is reduced by NO_2_ gas in traffic situations [92]. Most authors seek to calibrate their sensor networks using recalibration algorithms. These can be based on comparisons with artificial gases measured in the laboratory or on reliable real measurements taken at fixed and controlled stations [17,93,94].

The most recent studies are in favour of large networks of low-cost NDIR sensors. To achieve the desired stability of measurements, the instruments must be corrected at regular intervals with data from a reference instrument or control parameters to which such sensors are cross-sensitive (gas mixture, atmospheric pressure, temperature, or relative humidity). In addition, sensor-specific corrections are required and must be considered dependent on time, e.g., by including a linear offset that only becomes more evident for long-term observations [48]. 

According to the published results, the practical error of these sensors was reduced by <5ppm, or approximately 1% of the observed value for response times of 60 s, by means of individual and periodic recalibration for each of the sensors in the network, using algorithms that consider these parameters. For average response times of 200 s, measurement noise is reduced by up to 30% [90].

## 3. Gas Measurement in Tunnels

The control of gas concentration in tunnels is a matter of concern for all administrations involved in tunnel management. Real-time monitoring of the air quality inside the tunnel is essential in order to be able to regulate the operation of the ventilation system [21,22,23,26]. The volume of air extracted by this ventilation system must be one that maintains tolerable levels of gases without incurring high operating costs, which are mainly due to the amount of energy used during its operation. In addition, the continued use of exhaust fans leads to accelerated wear and tear of their moving parts, resulting in a shortened service life and, consequently, further costs associated with their replacement [23]. In order to lessen these costs, some researchers have researched the use of natural ventilation in long tunnels, which can lead to savings of up to 40% in smoke extraction costs [20,21,24]. According to some research, smoke extraction costs have been quantified as up to 75% of energy costs [20,21,22].

The measurement of this concentration is therefore a long-standing line of research. Table 2 shows a compilation of specific infrastructures in which research projects aimed at measuring gas concentration have been carried out.

The studies in Table 2 have been carried out in tunnels around the world. Almost all of these studies have been carried out using NDIR sensors, although not all of them specify the type of sensor or have been able to verify the commercial sensor model used. Similarly, in each of them, the target gas is different, with NO_x_, CO_x_ and particulate matter being the most common gases measured, as these are the gases with the greatest presence in transport pollution. 

**Figure 7:**
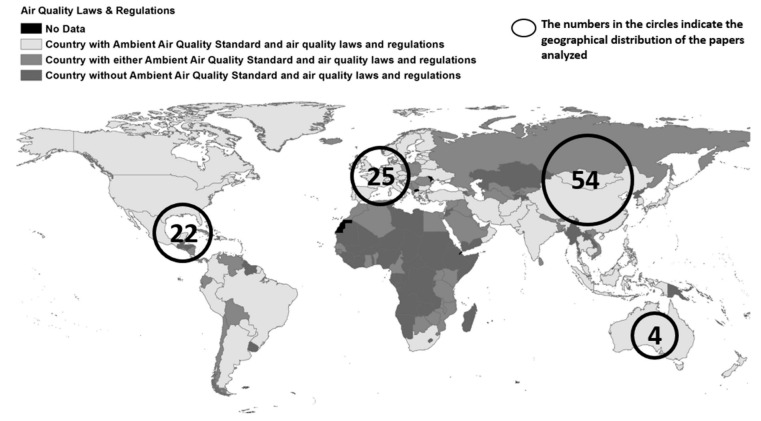
Map indicating global air quality laws and geographical distribution of studies [15].

A recent review of this research has been carried out by Marinello et al. [15], with more than 100 studies on tunnels of various types, tunnel sections and tunnel ventilation systems around the world. Figure 7 shows the distribution of the reviewed studies, demonstrating that these studies are generally carried out in countries with air quality regulations. Most of these articles relate to tunnels of about 2000 m in length, with four traffic lanes, with an average speed of 66 km/h and a location in urban environments. These tunnels are often associated with high traffic intensities and the accumulation of pollutants. In addition, the methods of dilution and pollutant gas extraction are critical for the FM of the tunnel and for the air quality in its surroundings. 

Almost all the structures studied in this article are equipped with systems of forced ventilation. The concentration level of specific pollutants is one of the main factors determining the activation of these systems, which were switched off during data collection and air pollutant analysis. Sampling campaigns varied in duration from a few hours to a few days, or even longer periods in the cases where there were seasonal effects on gas concentration levels. The measurement campaigns were generally carried out by placing the instrumentation in the tunnel portals or in the middle of the tunnel.

The types of pollutants analysed are very different and characteristic of each study. The most analysed pollutants are PM, CO, NO_2_ or CO_2_. However, the concentration values detected by different authors show considerable differences for all pollutants. Some average values reported are 237 µg/m^3^ for PM_10_, 107 µg/m^3^ for PM_2.5_ or 551 µg/m^3^ for NO_2_. On the other hand, CO is the reference gas often used for the activation of automatic ventilation systems that are switched on when pollutant levels exceed specific critical limits. The average CO concentration is 17.4 mg/m^3^.

Almost half of the studies also provide meteorological data, which were closely associated with their territory. Temperatures ranged from −4 °C to 36 °C and relative humidity varied from 40% to 88%. However, these data in each individual tunnel remained virtually constant. The average temperature was 26.5 °C, and humidity was between 63.0% and 88.3% inside the tunnel for mild urban environments [14,17,26]. 

On the other hand, this study only classifies the concentration measurement methods as passive, semi-active or active, according to whether or not the air is forced through a filter, and their analysis is carried out in the laboratory. Regarding the type of sensors used in some of the more recent studies, there is some research using electrochemical sensors to measure PM and polycyclic aromatic hydrocarbon (PAH) compounds [14], but infrared optical sensors are frequently used [7,8,9,10,11,12,13].

### Gas Measurement for Ventilation Indoors

In recent years, there has been a boom in the development of low-cost and portable sensors for the measurement of indoor CO_2_ concentration levels [2,47,124]. This has been the parameter used to determine the need for space ventilation [33]. NDIR sensors have generally occupied this market space and have even been used for more specific medical applications [125,126]. 

These low-cost sensors have many limitations in terms of sensitivity and stability. However, their use has become widespread and the number of studies on their use has proliferated [30]. Numerous recommendations have followed, mainly the development of regular maintenance and recalibration plans [127,128], as well as concentration prediction models [129,130,131,132,133].

## 4. Discussion and Future Developments

The measurement of combustion gases inside a road tunnel is a key factor in its proper FM. The volume of traffic or the concentration of several gases, such as CO or CO_2_, have been used to trigger the forced ventilation systems. A measurement of the concentration of these gases is essential to assess the operating level of the fans, whose operating costs due to energy consumption are a major item in the O&M of the infrastructure budgets [15,20,21,22].

There are a multitude of sensor types on the market, according to their approach to gas detection [27]. Electrochemical sensors based on solid oxide electrolytes are considered to have the most reliable method of detecting CO_x_ in industrial applications; however, they require very high operating temperatures of around 450 °C [29,50]. Therefore, such sensors can be discarded when measuring air quality inside a tunnel, where the average temperature is 26 °C. Having several such devices along the length of a road tunnel would increase costs and operational complexity. There are also electrochemical gas sensors in solution that are often used in specific engineering or medical applications [52,55]. Such sensors are characterized by their technical complexity, and their arrangement of a group of sensors along a tunnel would increase the operational constraints of the system. 

MOS-type sensors are a breakthrough in electrochemical sensors, with high sensitivity, a fast response time and stable performance [58]. Nevertheless, their selectivity is reduced in environments with high concentrations of other gases or humidity [28,50,62], often requiring a high operating temperature range. Lastly, MOFs sensors have different operating principles allowing a wide variety of devices that are adjusted according to the needs of each specific case, so these types of sensors has a promising future due to their sensitivity, wide operating temperature range and structural and chemical adaptability [66,73,77,81]. However, such sensors are still under development and would not be reliable in practical application.

The other main group are optical sensors, NDIR sensors being the most widely used in both domestic and industrial applications [39]. They have good sensitivity, operate at ambient temperature and have a long lifetime; however, they have a worse response time and lower sensitivity and selectivity compared to electrochemical sensors. Their use has become more extensive and cheaper with the development of low-cost sensors for domestic use [33,42,47], although their implementation is influenced by the presence of other gases or humidity [36,43,45]. Both selectivity and sensitivity can be improved by optics, but this means increasing the size of the device, increasing its cost, response time and power consumption [44]. However, the growing concern about indoor air quality has caused the proliferation of this type of device, increasing the variety of NDIR sensors in the market and lowering its costs [33].

Table 3 classifies the references cited in this article. If we look at the type of sensor, the most recent studies on practical applications are strongly influenced by the increase in demand for sensors for monitoring CO_2_ concentration in domestic indoor environments, such as tunnels or underground parking. In the case of infrastructures, it is notable that among the articles reviewed, almost all of those that specify the type of sensor or model used are of the NDIR type. This is also the case for monitoring the concentration of gases in the environment. This could be since in all three cases rapid measurements are required, at ambient temperature and low-cost and portable sensors. Moreover, in these cases a certain measurement error can be assumed, i.e., lower sensitivity and selectivity can generally be assumed. On the other hand, when very reliable measurements are needed in industrial processes, research tends to focus on electrochemical-type sensors, where the reactive components of the sensor can be determined to suit each singular process within the wide variety of materials available. In addition, under the electrochemical sensor type, a wide variety of sensing forms are grouped together, which accounts for the largest number of review articles analysed.

Reliable measurements are necessary for demand-controlled ventilation, but the environment in which the measurement is carried out is susceptible to changes in ambient conditions that lead to errors in the measurement, regardless of the type of sensor [32]. Although the humidity and temperature conditions inside the tunnel are kept almost constant [14,17,45,94], changes are produced during the course of both the day and the year by outdoor environmental conditions. These oscillations cause a drift in gas measurements that is only evident in long-term observations [48]. In the case of infrared sensors, an association between sensor age and measurement accuracy cannot be correlated, nor can inaccuracies in measurement be attributed to errors in the translation of the sensor’s output signal, but rather to a measurement bias of the sensor itself [91] or environmental conditions [32].

Therefore, a recalibration of the sensors is essential during their lifetime, regardless of the detection method. However, most demand-controlled ventilation infrastructures only carry out an initial calibration after installation and testing of the sensor [91]. The combination of all these factors produces inaccurate measurements, which may not be perceptible at the time. This eventually results in cost overruns during operation due to excessive fan use or deficits in the air quality inside the tunnel [23]. 

New research is focusing on extensive networks of low-cost NDIR sensors. The sensors are corrected at regular intervals with data from a reference instrument, and the parameters that are cross-sensitive for this type of sensor are jointly monitored. Each sensor is individually recalibrated, and the practical error is reduced to 1% of the observed value for a response time of 60 s [48,90]. 

Furthermore, there has been a proliferation of low-cost NDIR-type sensors to measure CO_2_ concentration levels in domestic environments over the last few years [33]. These environments are maintained at room temperature and at a relative humidity of around 50% [33,42,47]. These atmospheric parameters are similar to those found in urban road tunnels [14,17,26]. Therefore, research and advances in these types of sensors, interconnected and periodically recalibrated, can be extrapolated to the control of gas levels in tunnels where concentrations should not exceed 1000 ppm. In addition, advances in data processing have allowed the development of prediction models [128,129,130,131,132,133], able to anticipate the operating needs of the ventilation system and reduce the operating time. This represents a significant leap in reducing the operating costs of ventilation [23], and it can make a better adjustment in the activation of extraction systems using a network of sensors with lower capacity but with lower operational complexity and greater robustness to the environment to which they are exposed, i.e., the quality of the measure is improved. However, research has shown that maintenance and recalibration plans are needed for the use of such sensors [127,128]. 

A network of sensors connected to each other [22,134] and to the control centre provides the system with information, not only on the gas concentration measurement, but also on traffic data and environmental conditions (temperature, relative humidity and the presence of other gases). The smart processing of this data allows recalibration and demand prediction algorithms to be generated, thus optimizing the available resources, and reducing the operating costs of the tunnel ventilation system, thereby increasing the quality of the air inside the infrastructure.

## 5. Conclusions

Tunnel gas concentration monitoring is essential to preserve air quality without incurring high operating costs from the ventilation system. The following conclusions are drawn from this study:

- A mixture of pollutant gases emitted by traffic accumulates inside tunnels, leading to a deterioration of indoor and outdoor air quality, with harmful consequences for health and the structural integrity.
- Real-time monitoring with accurate measurements requires an efficient sensor system to provide continuous data readings to control the use of the ventilation system, without raising operational costs.
- Environmental conditions, mainly temperature, relative humidity and gas mix, influence the reading of the sensors, causing measurement deviations regardless of the detection method. Periodic recalibration of the sensors becomes essential to maintain error-free monitoring.
- MOS-type sensors have high sensitivity, but their lifetime and high operating temperature lead to high operating costs. New electrochemical sensors can cover the main needs, but they are still under development and would not be effective for a real application.
- Optic sensors are the most widely available on the market, with a broad range of value for money in relation to the operational needs, with a useful life of above 10 years. Many types of NDIR sensors have been developed with the proliferation of applications for domestic use, reducing the cost of this type of sensor.
- A network of low-cost NDIR sensors which are linked together take all other ambient and traffic factors into consideration. Periodic recalibration and processing of (the) data will allow for an efficient ventilation system, optimising the available resources in the tunnel management.

## Figures and Tables

**Table 1:** Comparison of various types of gas sensors [29,89].

Parameter	Type of Gas Sensors		
	Semiconductor	Electrochemical	Infrared Absorption
Sensitivity	E	G	E
Accuracy	G	G	E
Selectivity	P	G	E
Response time	E	P	P
Stability	G	B	G
Durability	G	P	E
Maintenance	E	G	P
Cost	E	G	P
Portable instrument	E	P	B
E: excellent, G: good, P: poor, B: bad			

**Table 2:** Compilation of tunnels in which research projects aimed at measuring gas concentration.

Infrastructure Name	Ref.
Annie Cordy Tunnel. Tunnel in Brussels city centre, Belgium	[95]
Strømsås Tunnel. Road tunnel in Drammen, Norway	[96]
Caldecott Tunnel. Tunnel in San Francisco Bay, California	[97]
Urban traffic tunnels in Lisbon, Portugal	[98]
Liberdade Avenue Tunnel (Braga, Portugal)	[46]
Grand Mare Tunnel. Road tunnel located in Rouen, France	[99]
Tingstad Tunnel. City tunnel in Gothenburg, Sweden	[100]
Washburn Tunnel. Urban tunnel in Houston, Texas	[101]
Cross Harbour Tunnel. Underwater urban tunnel in Hong Kong, China	[102]
Hsuehshan Tunnel. Road tunnel on the Taipei-Yilan Freeway, Taiwan	[103]
Gubrist tunnel. Highway tunnel in Zurich, Switzerland	[104]
Craeybeckx Tunnel. Highway tunnel in Antwerp, Belgium	[105]
Mount Bolu Tunnel. Highway tunnel in Turkey	[106]
Osmaganzi Tunnel. Highway tunnel located in Bilecik, Turkey	[107]
Highway tunnel in Pittsburgh, Pennsylvania	[108]
Kaisermühlen Tunnel. Urban tunnel in Vienna, Austria	[109]
Söderleds Tunnel. Urban tunnel in Stockholm, Sweden	[110]
Túneles de carretera en Pittsburgh, Pennsylvania	[8]
Xiangyin Tunnel. Urban tunnel in Shanghai, China	[111]
Yan’an East Road Tunnel. Urban tunnel in the centre of Shanghai, China	[112]
Buk-Ak Tunnel. Highway tunnel in Seoul, Korea	[113]
Loma Larga Tunnel. Highway tunnel located in Monterrey, Mexico	[12]
Fort McHenry Tunnel. Highway tunnel in Maryland	[11]
Tuscarora Tunnel. Highway tunnel in Pennsylvania	[11]
Marquês de Pombal Tunnel. Urban tunnel in Lisbon, Portugal	[13]
‘4 Giornate’ Tunnel. Urban tunnel in Naples, Italy	[114]
Westgate Tunnel. Leeds urban tunnel, United Kingdom	[115]
Wujinglu Tunnel. Urban tunnel in Tianjin, China	[116]
Tingstad Tunnel and Lundby Tunnel. Urban tunnels in Gothenburg, Sweden	[117]
Thiais Tunnel. Highway tunnel in Paris, Francia	[118]
Urban tunnels in Sao Paulo, Brasil	[119]
Xiamen XiangAn Tunnel. Undersea tunnel in China	[120]
Mountain tunnel in Nanjing, China	[121]
Kiesberg Tunnel. Highway tunnel located between Düsseldorf and Wuppertal	[122]
Domain Tunnel and Burnley Tunnel. Urban tunnels en Melbourne, Australia	[123]

**Table 3:** References classified according to application and sensor type.

Issue	NDIR Sensors	Electrochemical Sensors
review	[29,33,35,36,37,38,39,44,46]	[29,35,50,56,58,60,61,62,65,69,71,72,73,76,86,89]
domestic applications	[32,33,37,40,42,43,45,88,91,125,126,128,129,130,131,132,133]	[66,67,68,82,83,84,88]
industrial applications	[4,35,41,91,94]	[25,28,41,51,52,53,54,55,59,63,64,70,74,75,77,78,79,80,81,85,87]
infrastructure	[4,7,9,10,11,12,13,14,15,17,22,90,98,104,113,116,120,122]	[15,55]
environment	[5,6,9,30,31,35,48,93,94]	[5,6,27,55,57,92]

## Data Availability

The data presented in this study are available on request from the corresponding author.
